# Gene Expression-Based Predication of RNA Pseudouridine Modification in Tumor Microenvironment and Prognosis of Glioma Patients

**DOI:** 10.3389/fcell.2021.727595

**Published:** 2022-01-18

**Authors:** Lin-jian Wang, Peipei Lv, Yongli Lou, Jianping Ye

**Affiliations:** ^1^ Department of Neurosurgery, Zhengzhou Central Hospital Affiliated to Zhengzhou University, Zhengzhou, China; ^2^ Metabolic Disease Research Center, Zhengzhou Central Hospital Affiliated to Zhengzhou University, Zhengzhou, China; ^3^ Department of Radiology, Zhengzhou Central Hospital Affiliated to Zhengzhou University, Zhengzhou, China; ^4^ Center for Advanced Medicine, College of Medicine, Zhengzhou University, Zhengzhou, China

**Keywords:** glioma, pseudouridine, prognostic model, tumor microenvironment, inflammatory molecule

## Abstract

Aberrant expression of methyltransferases and demethylases may augment tumor initiation, proliferation and metastasis through RNA modification, such as m^6^A and m^5^C. However, activity of pseudouridine (Ψ) modification of RNA remains unknown in glioma, the most common malignant intracranial tumor. In this study, we explored the expression profiles of the Ψ synthase genes in glioma and constructed an efficient prediction model for glioma prognosis based on the CGGA and TCGA datasets. In addition, the risk-score signature was positively associated with malignancy of gliomas and the abundance of tumor-infiltrating immune cells such as macrophages M0 and regulatory T cells (Tregs), but negatively associated with the abundance of monocytes, NK cell activation and T cell CD4^+^ naive. In terms of mechanism, the risk-score signature was positively associated with the expression of inflammatory molecules such as S100A11 and CASP4 in glioma. Overall, this study provided evidence for the activity of RNA Ψ modification in glioma malignancy and local immunity.

## Introduction

More than 160 distinct chemical modifications have been identified in RNA molecules (Boccaletto et al., 2018), which may regulate RNA stability and function in eukaryotic cells. N^6^-methyladenosine (m^6^A) modification (Dominissini et al., 2012) is involved in regulation of a broad range of cellular activities, such as viral infection, vascular development, autoinflammatory disorders and cancer (Wang L. J. et al., 2020; Zhou et al., 2020; Tang et al., 2021). The study of m^6^A promotes investigation of other RNA modifications, such as 5-methylcytidine (m^5^C), N^1^-methyladenosine (m^1^A), N^7^-methylguanosine (m^7^G), ribose methylations (Nm), N^4^-acetylcytidine (ac^4^C) and pseudouridine (Ψ) modification (Wiener and Schwartz, 2021). These RNA modifications may directly affect the activities of mRNA in several ways, including decay, translation efficiency and stability, and thus shape the transcriptomic landscapes (Frye et al., 2018). *Ψ* is a prevalent type of nucleoside modification that occurs in both non-coding RNA and mRNA (Carlile et al., 2014). *Ψ* plays an essential role in various molecular mechanisms, such as the stabilization of RNA structure (Davis et al., 1998; Charette and Gray, 2000), RNA-RNA and RNA-protein interactions (Basak and Query, 2014) and the metabolism of RNAs (Ma et al., 2003; Carlile et al., 2014). Pseudouridylation is catalyzed by pseudouridine synthases. In eukaryotic cells, there are 17 different pseudouridine synthases, while 12 pseudouridine synthase genes have been discovered in humans: PUS1, PUS3, PUS7, PUS7L, PUS10, DKC1, RPUSD1, RPUSD2, RPUSD3, RPUSD4, TRUB1, and TRUB2 (Penzo et al., 2017). The abnormality of Ψ modification is associated with various diseases. For instance, DKC1 stabilizes the mRNA of some ribosomal proteins depending on its pseudouridine synthase activity, thereby promoting colorectal cancer progression *in vitro* and *in vivo* (Kan et al., 2021). Increasing evidences have suggested an association between abnormal expression of Ψ synthases (such as PUS1, PUS7, PUS10 and DKC1) and tumor malignant progression (Jana et al., 2017; Elsharawy et al., 2020; Du et al., 2021; Stockert et al., 2021). In addition, Ψ synthase inhibitors have been shown to repress tumor growth (Gmeiner, 2020; Kan et al., 2021). However, the activity of Ψ synthases remains unclear in glioma.

Primary gliomas are graded from I to IV according to the classification scheme specified by the World Health Organization (WHO) (Ostrom et al., 2018). WHO grade II and III gliomas, including astrocytomas, oligodendrogliomas and mixed oligoastrocytomas, are defined as lower-grade gliomas (LGG). WHO grade IV gliomas (Glioblastoma, GBM) are highly malignant, and its characteristics are significantly different from those of LGG (Ostrom et al., 2019). A wide range of efforts have been made during the past decades to improve the diagnosis and prognosis of glioma. Notably, RNA modification, especially m^6^A and m^5^C modification, have been proposed as a new class of epigenetic markers in the diagnosis of glioma malignancies, and the related enzymes have shown a great value in prognostic of glioma (Wang P. et al., 2020; Lin et al., 2020). However, there is no report on the relationship between *Ψ* synthase gene expression and characteristics of glioma.

In this study, we explored the expression profiles of Ψ synthases and found abnormal expression of seven synthase genes (PUS1, PUS7, RPUSD1, RPUSD3, DKC1, TRUB1 and PUS7L) in GBM relative to LGG in the CGGA dataset. The Ψ synthase genes that are closely related to overall survival (OS) were extracted to perform the least absolute shrinkage and selection operator (LASSO) multivariate Cox regression algorithm. Five positive genes (PUS1, PUS7, RPUSD1, DKC1 and TRUB1) were selected to construct a high efficacy prediction model. We identified differentially expressed genes (DEGs) between the high-risk group and the low-risk group to explore the mechanism by which risk-score signature affect prognosis. The results of GO analysis suggested that the up-regulated DEGs were significantly enriched in the signaling pathway of immune-related reactions. Moreover, analysis of immune landscape suggested that the tumor microenvironment (TME) and tumor-infiltrating lymphocytes (macrophages M0, monocytes, NK cell activation, T cell CD4^+^ naive and Tregs) were significantly different between the high-risk group and low-risk group. Weighted correlation network analysis (WGCNA) was applied to identify the candidate hub genes that may regulate TME and immune cell infiltration of glioma based on the CGGA dataset. Finally, we found the expression of inflammatory molecules CASP4 and S100A11 were positively associated with Ψ synthase-related risk-score signature. In summary, we identified the link of Ψ synthase genes to glioma prognosis, and potential value of Ψ modification in glioma malignancy and local immunity.

## Methods and Materials

### Datasets and Samples

The Chinese Glioma Genome Atlas (CGGA) RNA-seq data (#325) and clinical data were downloaded from the CGGA data portal (http://www.cgga.org.cn/) as the training set. The merged GBMLGG datasets (The Cancer Genome Atlas, TCGA) were downloaded from the cBio Cancer Genomics Portal (cBioPortal, http://www.cbioportal.org) as the validation set (Cerami et al., 2012). After excluding samples with missing clinical data such as survival and WHO grade, 309 (CGGA dataset) and 674 (TCGA dataset) glioma patients were finally enrolled in this study. In addition, the CGGA RNA-seq data (#693), CGGA mRNA array data and the GSE59612 expression data were downloaded from CGGA data portal and GEO website (https://www.ncbi.nlm.nih.gov/geo/), respectively.

Five WHO grade II/III and five WHO grade IV glioma samples were collected from patients undergoing surgical treatment from November 2019 to December 2020 in Zhengzhou Central Hospital Affiliated to Zhengzhou University. The clinical diagnosis was confirmed by immunohistochemical staining. This study was approved by the institutional review board of Zhengzhou Central Hospital Affiliated to Zhengzhou University, and informed consents were obtained from all patients.

### Construction of the Risk Score Model

The difference in Ψ synthase gene expression between LGG and GBM was identified in the datasets of CGGA, TCGA and GSE59612. Univariate Cox regression analysis was performed to identify the genes related to overall survival (OS). Five Ψ synthase genes were screened out by performing the least absolute shrinkage and selection operator (LASSO) multivariate Cox regression algorithm in the R package “glmnet”. Finally, the signature genes and coefficients in the risk-score signature were constructed based on the most suitable penalty parameter λ. The risk score formula used in this study was:

$$Risk\;score=\overset{n}{\underset{i=1}{\sum }}\left(Coef_{i}\text{∗}Exp_{i}\right)$$

where $Coef_{i}$ is the coefficient, and $Exp_{i}$ is the normalized expression of each signature gene.

### Identification of DEGs and GO Enrichment Analysis

The differentially expressed genes (DEGs) were identified by executing the “limma” package in R software (Ritchie et al., 2015). EDGs were determined with adjusted *p*-value < 0.05 and |log_2_FC| ≥ 1 in fold change between the high-risk and low-risk groups in the CGGA and TCGA datasets, respectively. Gene Ontology (GO) enrichment analysis was conducted using the R package “clusterprofiler” (Yu et al., 2012).

### Immune Landscape Analysis

The immune scores, stromal scores and ESTIMATE scores of gliomas were calculated using the “estimate” package in R software. The Immunophenoscores of gliomas were calculated using IPS algorithm (Charoentong et al., 2017). The abundance of tumor-infiltrating immune cells were evaluated on the TIMER2 platform (http://timer.cistrome.org/) based on gene expression profiles (Li et al., 2020).

### Weighted Correlation Network Analysis (WGCNA)

In order to identify the clinical traits-related modules and hub genes, DEGs identified in both CGGA and TCGA datasets and clinical traits were incorporated to perform Weighted correlation network analysis (WGCNA) using R package “WGCNA”. The adjacency matrix was then transformed into topological overlap matrix (TOM). Genes were divided into different gene modules according to the TOM-based dissimilarity measure. The soft-thresholding power was set at five in the scale-free network. Hub genes of each module were computed and the close modules were integrated with setting the height  to  0.25, the deep split to two and the min module size  to 20.

### Immunohistochemical Staining

After deparaffinization and rehydration, the tissue slices were submerged in the peroxidase blocking solution which was mixed with one part 30% hydrogen peroxide and nine parts methanol for 10 min. After that, the slices were treated with 0.01 M citrate buffer and incubated with serum blocking solution for 20 min, and followed by incubation with anti-DKC1 (Abcam, ab156877, 1:100) or anti-RPUSD1 (Merck, HPA041144, 1:500) primary antibody overnight at 4°C. Thereafter, the slices were incubated with biotin-labeled secondary antibody for 20 min, and followed by incubation with horseradish peroxidase-conjugated avidin for 20 min. Finally, the sections were stained with diaminobenzidine, and nuclei were counterstained with hematoxylin.

### Statistical Analysis

One-way ANOVA, wilcox test, and *t* test were used to identify significant difference in gene expression and infiltration of immune cells in gliomas with different WHO grades and different risk groups. Univariate, multivariate, LASSO Cox regression and Kaplan-Meier analyses were performed to construct and evaluate the risk signature using the R packages “glmnet” and “survival”. Roc curve analysis was performed to predict the OS of glioma patients through the R package “survivalROC”. All statistical analyses were conducted using GraphPad Prism six software, R software v4.0.1 and SPSS v26, and a *p* value of less than 0.05 was considered statistically significant.

## Results

### Identification of Pseudouridine Synthase Genes in Glioma

**FIGURE 1:**
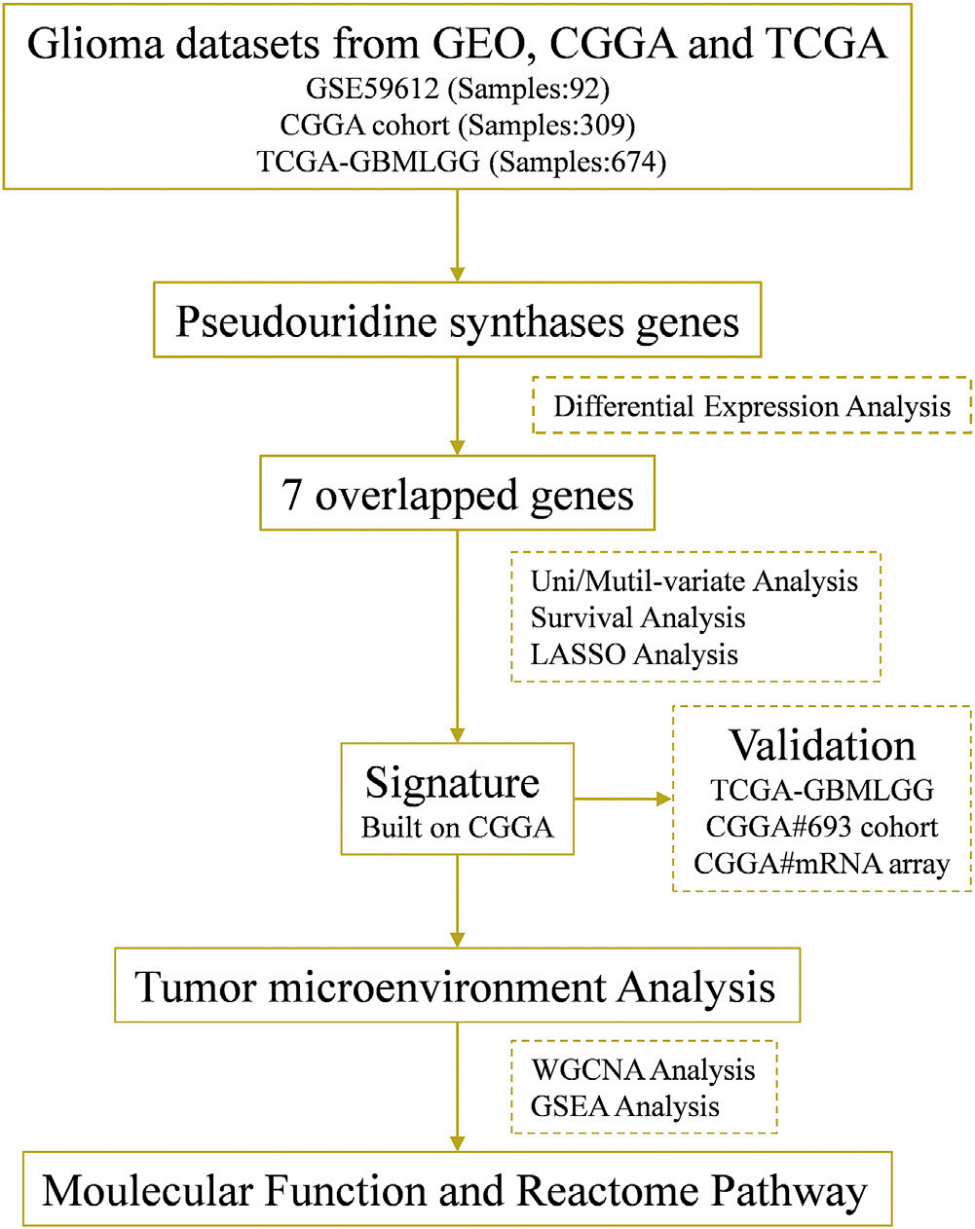
Flow chart of this study.

**FIGURE 2:**
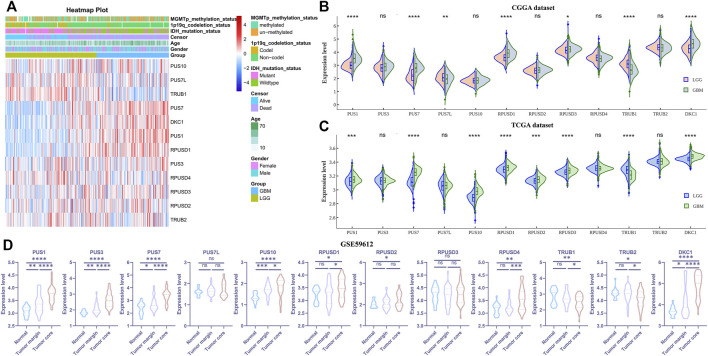
The expression levels of the 12 pseudouridine synthase genes in glioma **(A)** Heatmap depicting the expression levels of the 12 pseudouridine synthase genes in the CGGA dataset **(B–C)** Violin plot showing the expression levels of the 12 pseudouridine synthase genes between LGG and GBM in the CGGA and TCGA datasets, respectively **(D)** Violin plot showing the expression levels of the 12 pseudouridine synthase genes between paracancerous tissue (n = 17), tumor marginal tissues (n = 36) and tumor core tissue (n = 39) in the GSE59612 dataset. *p* values were calculated using wilcox test **(B–C)** and Student’s t-test **(D)**. *, *p* < 0.05; **, *p* < 0.01; ***, *p* < 0.001, ****, *p* < 0.0001.

Abnormal expression of certain Ψ synthases can affect the phenotype of cancer cells and behavior of tumor progression (Jana et al., 2017; Elsharawy et al., 2020; Du et al., 2021). As illustrated in the flowchart (Figure 1), we first analyzed the RNA-seq data of the CGGA, TCGA and GSE59612 datasets to characterize the expression pattern of 12 Ψ synthase genes in glioma. In the CGGA cohort, compared with LGG, the expression of PUS1, PUS7, RPUSD1, RPUSD3 and DKC1 were distinctly up-regulated in GBM; meanwhile, the expression of TRUB1 and PUS7L were significantly down-regulated in GBM (Figures 2A,B). In the TCGA cohort, except that PUS7L was not differentially expressed, the expression of other genes between LGG and GBM was similar to that of CGGA dataset (Figure 2C). In the GSE59612 dataset, the expression of PUS1, PUS7, RPUSD1 and DKC1 were significantly increased in the tumor core tissues relative to tumor marginal tissues and paracancerous tissues; and the expression of TRUB1 was decreased from paracancerous tissue to tumor core tissue (Figure 2D). Furthermore, the expression levels of these seven genes were significantly different in the groups compartmentalized by IDH-mutant status, MGMT promoter status, 1p/19q codel status and age in the CGGA dataset (Supplementary Figure S1).

### Construction of the Risk-Score Signature

**FIGURE 3:**
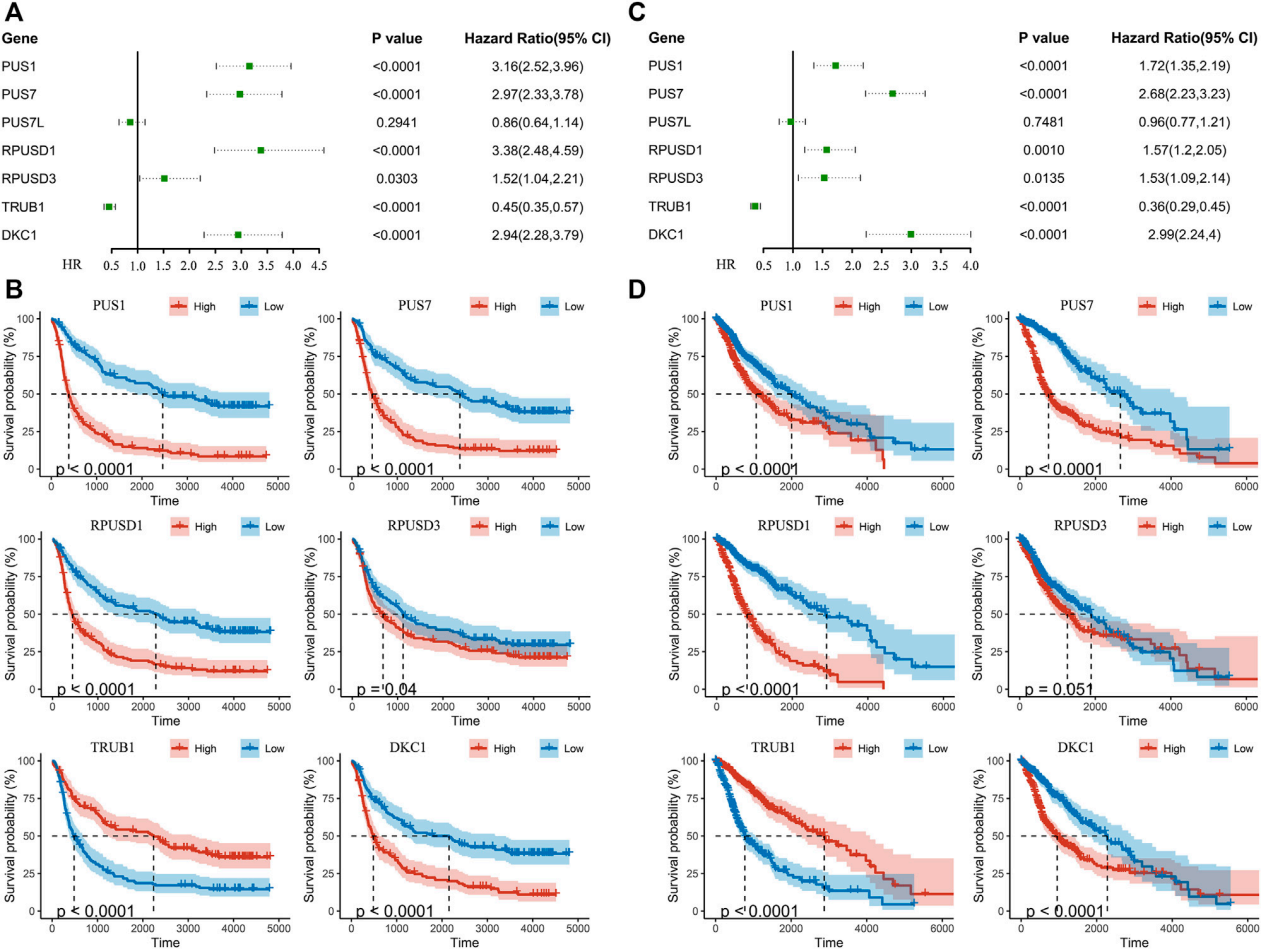
Identification of pseudouridine synthase genes that correlate with overall survival **(A)** Univariate Cox regression analysis of seven pseudouridine synthase genes in the CGGA dataset **(B)** Kaplan-Meier overall survival curves of certain genes in the CGGA dataset **(C)** Univariate Cox regression analysis of seven pseudouridine synthase genes in the TCGA dataset **(D)** Kaplan-Meier overall survival curves of certain genes in the TCGA dataset.

Univariate Cox regression analysis was performed to identify relationship between Ψ synthase genes and patient survival in the CGGA and TCGA datasets. Six Ψ synthase genes were associated with the prognosis, of which the expression of PUS1, PUS7, RPUSD1 and RPUSD3 was positively associated with the malignancy of glioma (HR > 1), and TRUB1 was negatively associated with the malignant grade (HR < 1) (Figure 3). Moreover, univariate Cox regression analysis was performed in different subgroups stratified according to pathological grade and IDH status. The results showed that PUS1, PUS7, and DKC1 were significantly correlated with the prognosis of gliomas in different subgroups (Supplementary Table S1).

**FIGURE 4:**
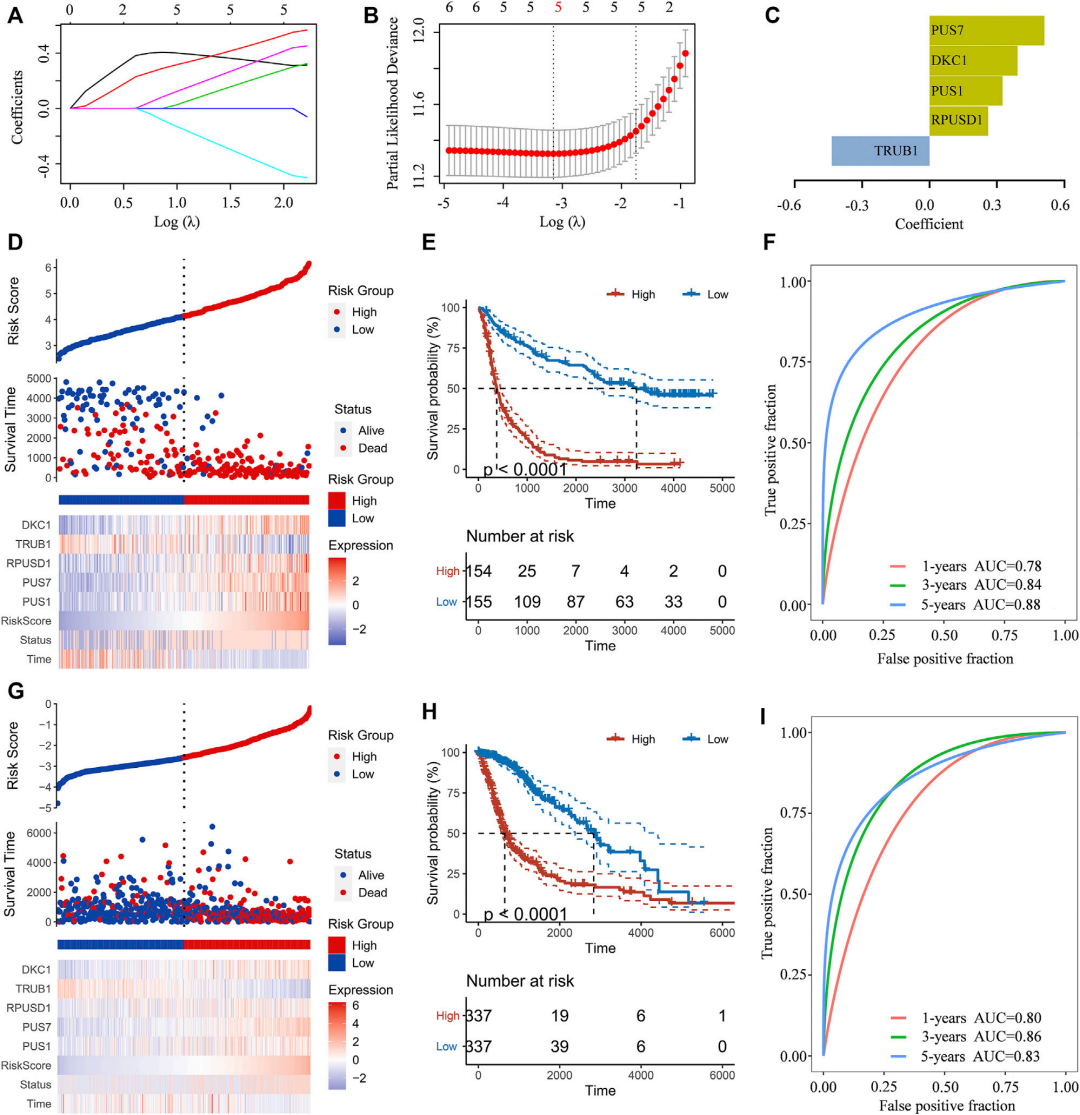
Construction of the risk-score signature using five pseudouridine synthase genes **(A)** LASSO coefficient profiles of the six pseudouridine synthase genes in the CGGA dataset **(B)** Partial likelihood deviance of different numbers of variables revealed by the LASSO regression model **(C)** Barplot displaying the coefficients constructed using the LASSO method **(D)** Distribution of risk score, patients’ survival status, and expression pattern of the five pseudouridine synthase genes in the CGGA dataset **(E)** Kaplan-Meier curves of overall survival according to risk score in the CGGA dataset **(F)** ROC curves showing the sensitivity and specificity of risk score in predicting the 1-, 3- and 5-years survival of glioma patients in CGGA dataset **(G)** Distribution of risk score, patients’ survival status, and expression pattern of the five pseudouridine synthase genes in the TCGA dataset **(H)** Kaplan-Meier curves of overall survival according to risk score in the TCGA dataset **(I)**. ROC curves showing the sensitivity and specificity of risk score in predicting the 1-, 3- and 5-years survival of glioma patients in TCGA dataset.

To predict the clinical outcomes of gliomas, the least absolute shrinkage and selection operator (LASSO) Cox regression algorithm was subsequently used to analyze the six Ψ synthase genes in the CGGA dataset (Figures 4A–C). Finally, five genes were screened out to construct the risk-score signature based on the minimum criteria in the training dataset (CGGA) (Figure 4D), and the signature was verified in the TCGA dataset, CGGA RNA-seq (#693) and CGGA mRNA array dataset (Figure 4G **,** Supplementary Figure S2). In order to assess the difference of overall survival between the low-risk and high-risk glioma patients, Kaplan-Meier analysis was performed both in the CGGA dataset and TCGA dataset. The results showed that the OS of glioma patients in the high-risk group was much shorter than that in low-risk group (Figures 4E,H, Supplementary Figure S2). Thereafter, ROC curve analysis was performed to assess the sensitivity and specificity of risk score in prediction of the 1-, three- and 5-years survival of glioma patients. The results showed that the risk score had high accuracy in the prediction of OS of glioma patients in the CGGA and TCGA datasets (Figures 4F,I, Supplementary Figure S2).

Considering age, WHO grade, IDH mutation status and 1p/19q coding status are related to the prognosis of gliomas, we analyzed the risk score signature in different subtypes stratified according to these pathological conditions. As shown in Supplementary Figure 3A, the risk scores of gliomas in GBM, IDH wild-type, older and 1p/19q non-coding subtypes were significantly higher than the corresponding subtypes. In addition, risk-score signature also exhibited high prognostic value in different WHO grade, IDH-mutant status, age and 1p/19q coding status subtypes (Supplementary Figure S3B–E).

Univariate and multivariate Cox regression analysis was performed to investigate whether the risk score was an independent prognostic factor for glioma. The results of univariate analysis showed that risk score, age, WHO grade and IDH status were significantly correlated with prognosis (Supplementary Figure S4A). Multivariate analysis also determined that risk score was significantly related to prognosis (Supplementary Figure S4B). A survival nomogram prediction model was then built based on independent prognostic parameters for the OS of glioma patients in the CGGA dataset (Supplementary Figure S4C). In addition, the calibration curves for the probability of 1-, three- and 5-years survival displayed excellent agreement between observation and prediction in the CGGA dataset (Supplementary Figure S4D). These results suggested that the risk-score signature of the five Ψ synthase genes was a reliable and independent prognostic indicator for gliomas.

### Verify the Expression of the Prognostic Ψ Synthase Genes

**FIGURE 5:**
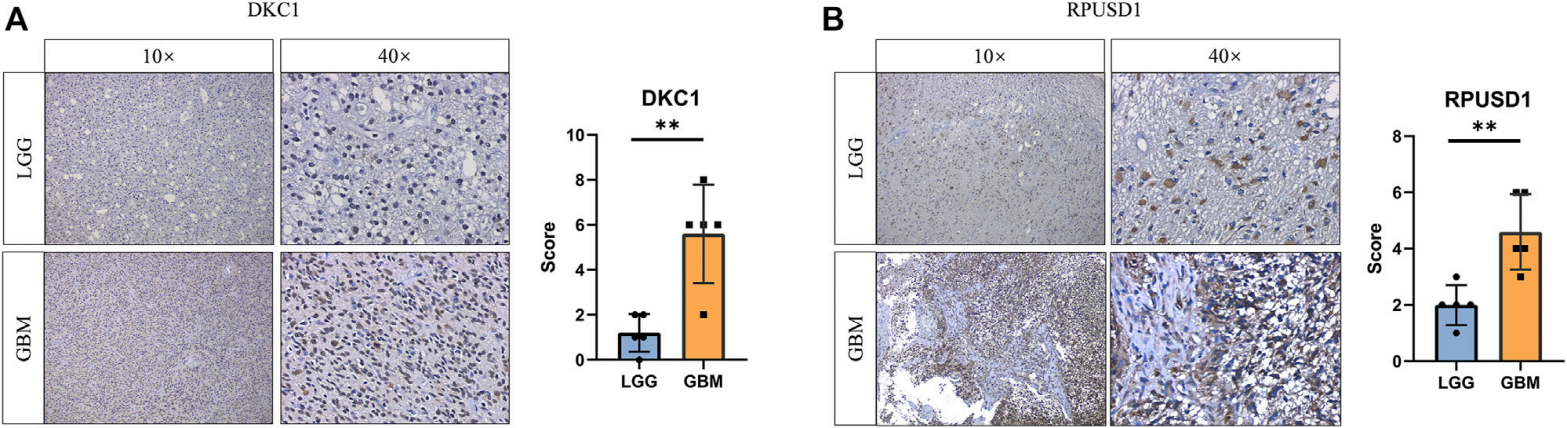
Validation the expression of prognostic pseudouridine synthase genes **(A–B)** Immunohistochemical staining analysis of the protein levels of DKC1 and RPUSD1 between the low-grade (n = 5) and high-grade gliomas (n = 5). *p* values were calculated using Student’s t-test. *, *p* < 0.05; **, *p* < 0.01; ***, *p* < 0.001.

To validate the expression of the prognostic Ψ synthase genes in glioma, we performed immunohistochemical staining on tissue samples collected from glioma patients undergoing surgical treatment. As shown in Figure 5, the expression levels of RPUSD1 and DKC1 in high-grade gliomas were higher than that in low-grade gliomas.

### Identification and Enrichment Analysis of DEGs Between High-Risk and Low-Risk Groups

**FIGURE 6:**
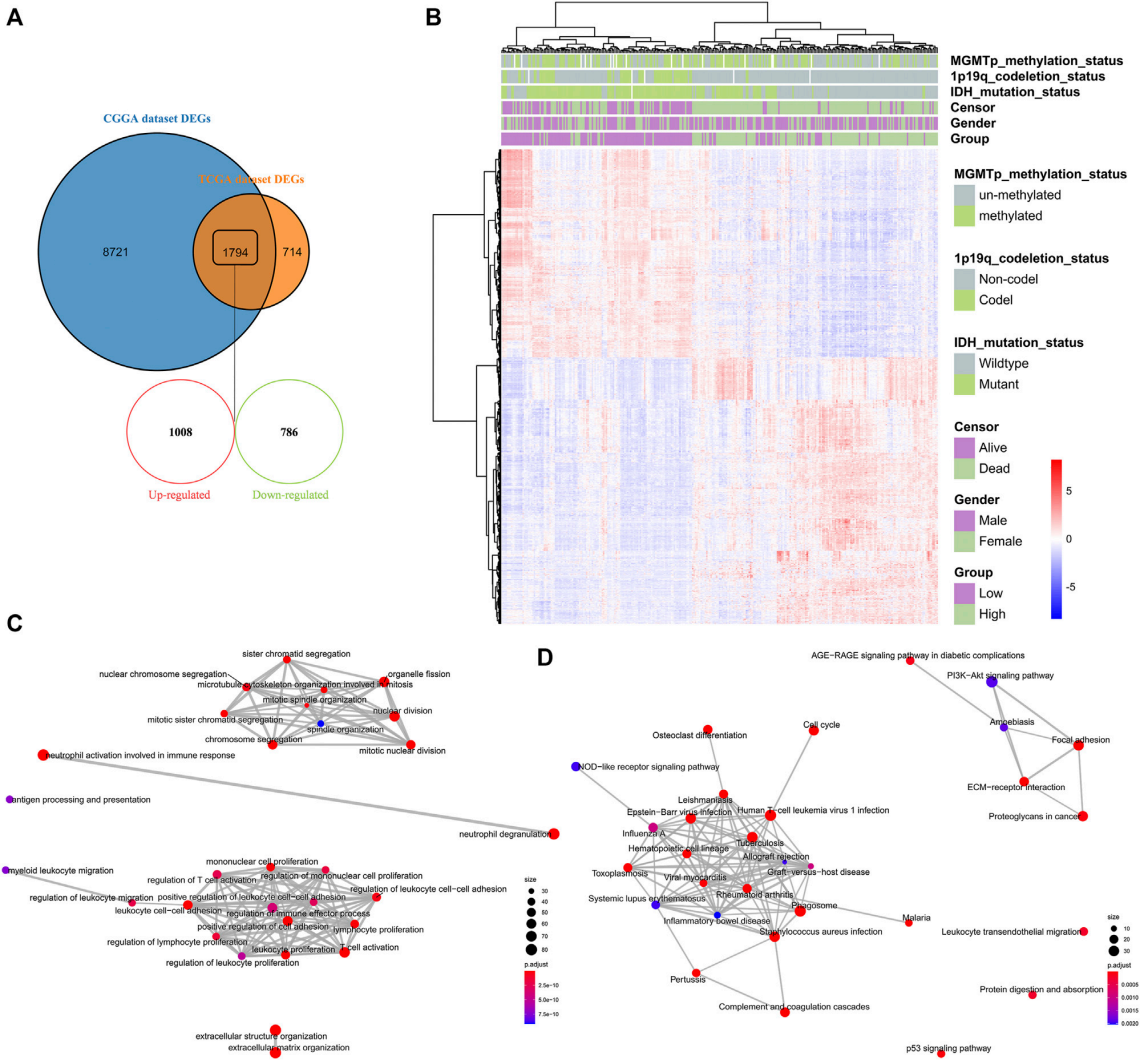
Identification and enrichment analysis of DEGs between high-risk and low-risk groups **(A)** Venn diagram showing the differentially expressed genes between the high-risk and low-risk groups in the CGGA and TCGA datasets **(B)** Heatmap depicting 1794 selected differentially expressed genes between the high-risk and low-risk groups in the CGGA dataset **(C–D)** GO analysis of the 1,008 up-regulated genes and 786 down-regulated genes, respectively.

To understand the impact of risk-score signature in the prognosis, we used R package “limma” to screen DEGs between the high-risk and low-risk groups. In the CGGA and TCGA datasets, 10,515 and 2,508 DEGs were screened, respectively (Figure 6A). 1794 genes that could be identified in both two datasets, including 1,008 up-regulated genes and 786 down-regulated genes, were selected for GO enrichment analysis (Figures 6A,B). Notably, the up-regulated genes were significantly enriched in immune-related signaling pathways such as the regulation of immune effector process, the regulation of T cell activation, the regulation of cell adhesion, and the mononuclear cell proliferation signaling pathway (Figures 6C,D).

### Immune Microenvironment Landscape of Glioma

**FIGURE 7:**
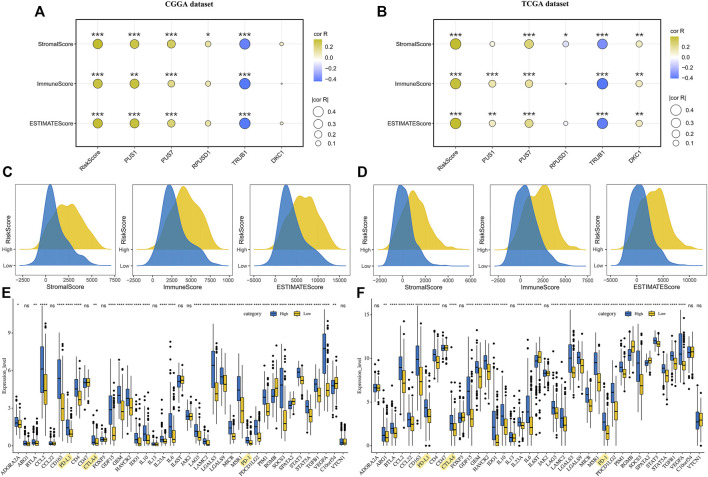
Correlation analysis of risk-score and immune microenvironment in glioma **(A–B)** Correlation analysis of risk-score and five pseudouridine synthase genes with immune scores, stromal scores and ESTIMATE scores in the CGGA and TCGA datasets, respectively **(C–D)** Ridge plot showing the distribution of immune scores, stromal scores and ESTIMATE scores of different risk groups in the CGGA and TCGA datasets, respectively **(E–F)** Boxplot displaying the expression levels of 39 immune signature genes in the CGGA and TCGA datasets, respectively. *, *p* < 0.05; **, *p* < 0.01; ***, *p* < 0.001.

The DEGs between the high-risk and low-risk groups were enriched in the immune-related signaling pathways, suggesting that the risk scores might be related to the tumor immune microenvironment. To test the possibility, we analyzed the distribution of immune scores, stromal scores and ESTIMATE scores in the groups with different risk scores. The results revealed that the higher risk scores were significantly correlated with the higher immune scores, stromal scores and ESTIMATE scores, respectively (Figures 7A–D). Moreover, correlation analysis suggested that the expression levels of the five prognostic Ψ synthase genes were associated with the immune scores, stromal scores and ESTIMATE scores, especially TRUB1 (Figures 7A,B). Immunophenotype were analyzed by IPS algorithm, the IPS scores of the high-risk group were lower than that of the low-risk group (Supplementary Figure 5), indicating the immunogenicity of gliomas in high-risk group was reduced. We examined the expression of immune-related genes, and found the expression of most immunosuppressive genes in the high-risk group was up-regulated, including the checkpoint genes such as PD-1, PD-L1 and CTLA4 (Figures 7E,F). Therefore, the Ψ synthase genes and risk-score might be related to the immunosuppressive microenvironment of glioma.

**FIGURE 8:**
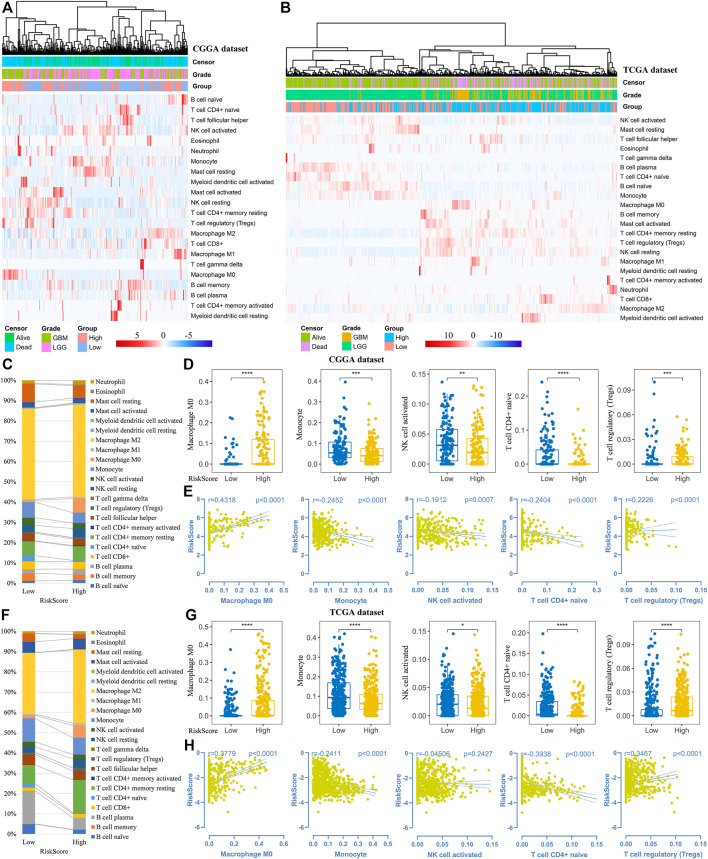
Analysis of immune cell infiltration in different risk-score groups **(A–B)** Heatmap depicting the infiltration levels of 22 immune cells in the CGGA and TCGA datasets, respectively **(C,F)** The average frequencies of 22 immune cells in low-risk and high-risk groups in the CGGA and TCGA datasets, respectively **(D,G)** Boxplot displaying the infiltration levels of macrophages M0, monocytes, NK cell activation, T cell CD4^+^ naive and Tregs between the high-risk and low-risk groups in the CGGA and TCGA datasets, respectively **(E,H)** Correlation analysis of risk-score with the infiltration levels of macrophages M0, monocytes, NK cell activation, T cell CD4^+^ naive and Tregs in the CGGA and TCGA datasets, respectively. *p* values were calculated using wilcox test. *, *p* < 0.05; **, *p* < 0.01; ***, *p* < 0.001, ****, *p* < 0.0001.

**FIGURE 9:**
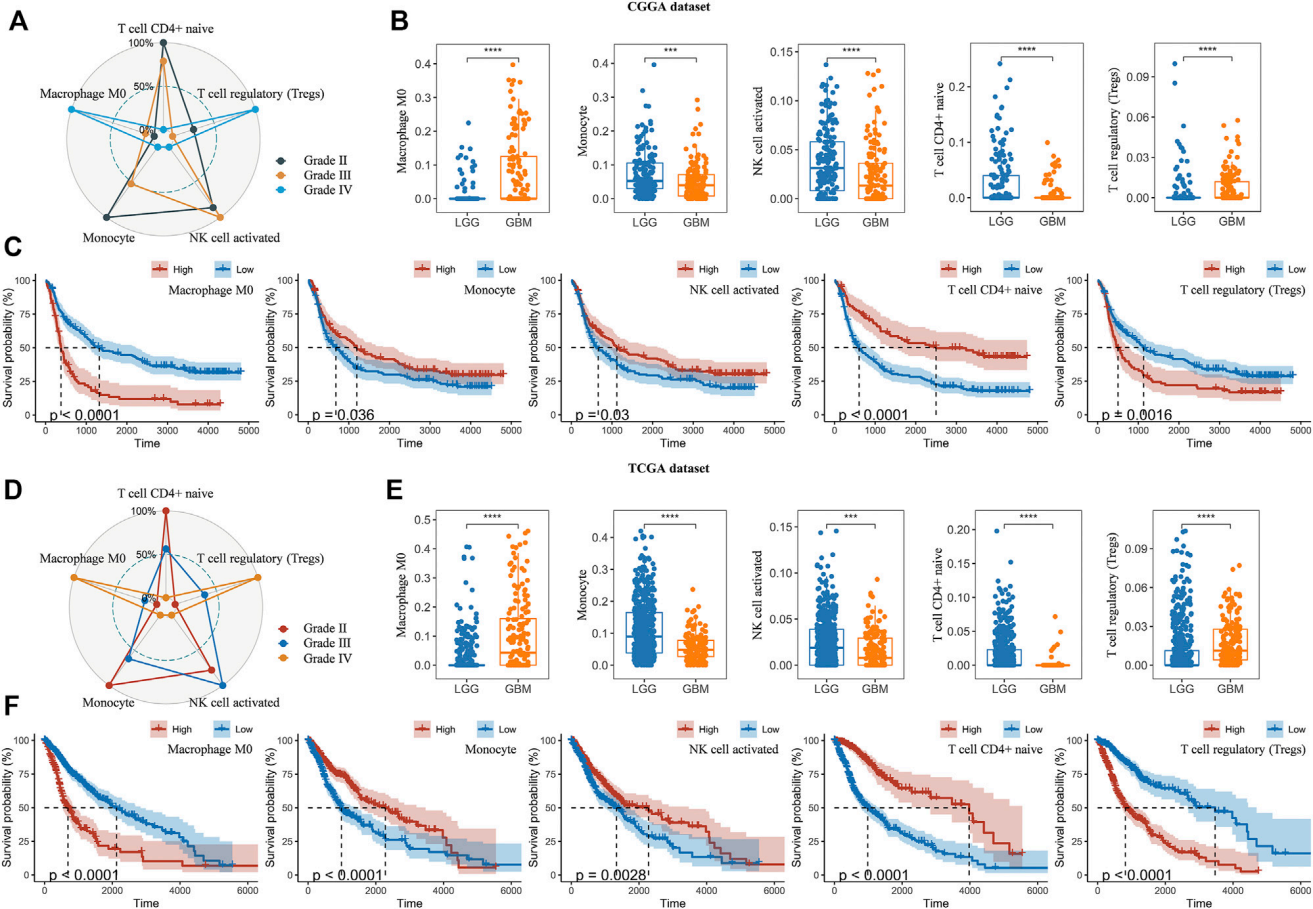
Analysis of immune cell infiltration in different WHO grades **(A,D)** Radar chart depicting the infiltration levels of macrophages M0, monocytes, NK cell activation, T cell CD4^+^ naive and Tregs between different WHO grades in the CGGA and TCGA datasets, respectively **(B,E)** Boxplot displaying the infiltration levels of macrophages M0, monocytes, NK cell activation, T cell CD4^+^ naive and Tregs between LGG and GBM in the CGGA and TCGA datasets, respectively **(C,F)** Kaplan-Meier overall survival curves of the infiltration levels of macrophages M0, monocytes, NK cell activation, T cell CD4^+^ naive and Tregs in the CGGA and TCGA datasets, respectively. *p* values were calculated using wilcox test. *, *p* < 0.05; **, *p* < 0.01; ***, *p* < 0.001, ****, *p* < 0.0001.

Thereafter, we investigated the association of risk scores and immune infiltration in CGGA and TCGA datasets. Twenty-two types of immune cells were analyzed using the CIBERSORT algorithm in the online tool TIMER2 (Figures 8A,B). The results suggested that the tumor infiltrating leukocytes including macrophages M0, monocytes, NK cell activation, T cell CD4^+^ naive and T cell regulatory (Tregs) were significantly different in number between the high-risk and low-risk groups. In detail, the infiltration of M0 and Tregs were increased in the high-risk group, while the infiltration of other cell types was decreased (Figures 8C,D,F,G). Based on the correlation analysis, we found the five types of immune cells were significantly associated with the risk scores (Figures 8E,H) and the expression of the five prognostic Ψ synthase genes (Supplementary Figure S6). As shown in Figures 9A,D, there were also significant differences in the infiltration levels of these five immune cells in different WHO grades. Similar to the comparison between different risk-score groups, the infiltration levels of M0 and Tregs increased in high grade gliomas, while the infiltration levels of the other three immune cells were significantly reduced in high grade gliomas (Figures 9B,E). Moreover, Kaplan-Meier survival analysis revealed that high infiltration levels of M0 or Tregs, and low infiltration levels of monocytes, NK cell activation or T cell CD4^+^ naive were associated with poor prognosis and low survival rates of gliomas (Figures 9C,F). In addition, the immune cell infiltration of different risk groups was analyzed in LGG and GBM subtypes, respectively. The infiltration levels of M0 and Tregs increased in high-risk gliomas, while the infiltration levels of other immune cells were significantly reduced in high-risk gliomas (Supplementary Figure S7).

### WGCNA and Module Analysis

**FIGURE 10:**
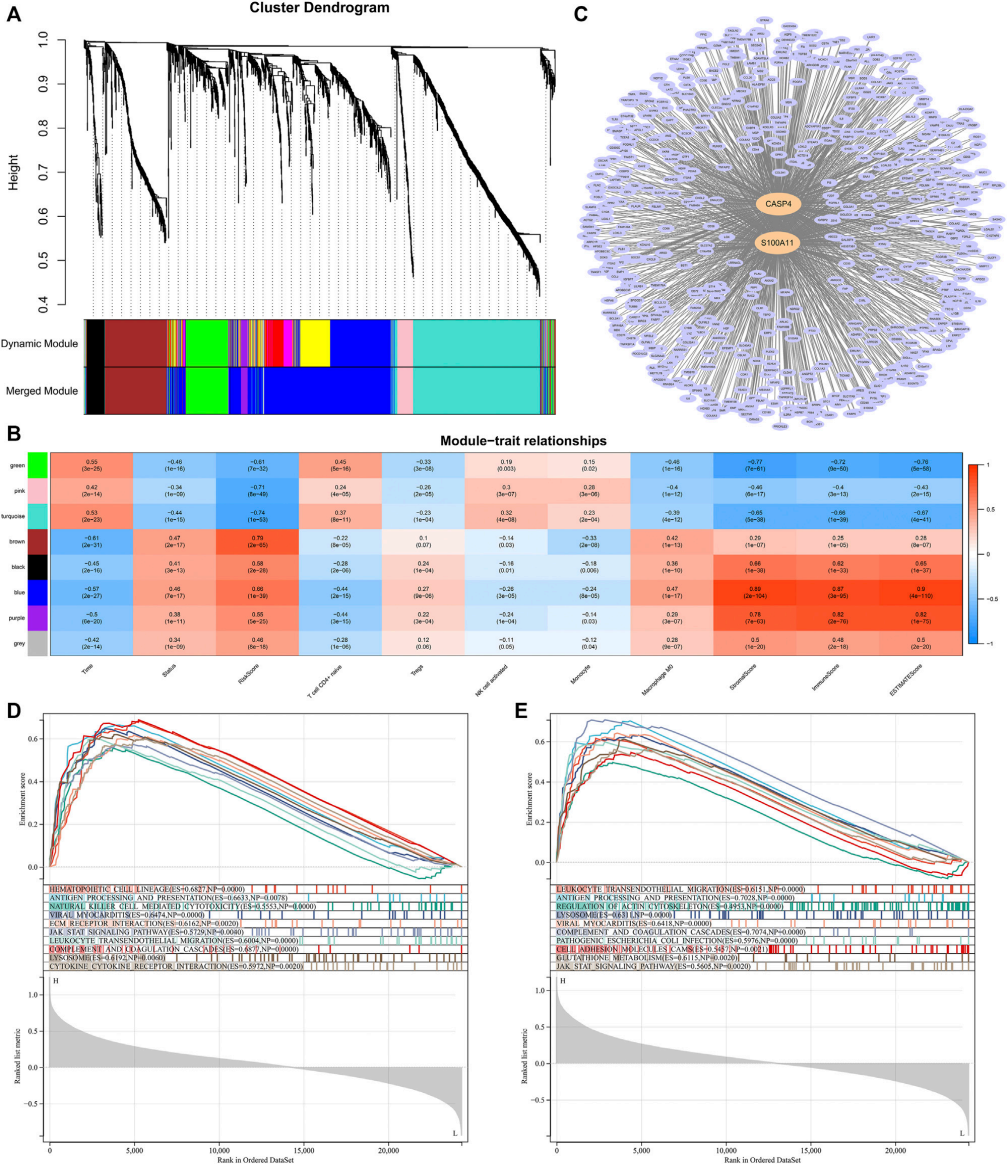
WGCNA and Module Analysis **(A)** Dendrogram of the differentially expressed genes clustered with dissimilarity measure based on topological overlap **(B)** Heatmap of the correlation between module eigengenes and clinical traits **(C)** Cytoscape analysis of the intersecting genes in the blue module **(D)** GSEA analysis based on the expression of CASP4 in the CGGA dataset **(E)** GSEA analysis based on the expression of S100A11 in the CGGA dataset.

We performed WGCNA to determine the hub genes that regulated TME and immune infiltration. The expression profile of 1794 DEGs and clinical traits of the CGGA samples were incorporated to construct a gene co-expression network. After merging similar modules with a threshold of 0.25, a total of eight modules were identified from the co-expression network (Figure 10A). As shown in the heatmap of module-trait relationships, the blue module was most relevant to clinical traits, especially traits such as immune scores, stromal scores, ESTIMATE scores, macrophages M0 infiltration (Figure 10B). In addition, two hub genes, CASP4 and S100A11, were identified from the blue module by setting the correlation relevant threshold to 0.9 (Figure 10C). Correlation analysis showed that the expression levels of CASP4 and S100A11 were correlated with the expression levels of the five prognostic Ψ synthase genes, and were significantly related to the infiltration levels of M0, monocytes, NK cell activation, T cell CD4^+^ naive and Tregs (Supplementary Figure S8, 9). Gene set enrichment analysis (GSEA) was further used to explore potential functional mechanisms or immunological associations of hub genes, the immune pathways such as antigen processing and presentation, leukocyte transendothelial migration, natural killer cell mediated cytotoxicity, JAK STAT signaling pathway, cytokine receptor interaction were significantly enriched (Figures 10D,E). Therefore, the risk-score signature might affect the expression of CASP4 and S100A11, resulting in changes in TME and the infiltration levels of the five immune cells.

## Discussion

Glioma is the most common malignant tumor in the brain of adults, and is considered one of the most devastating cancers due to the aggressive behavior and lack of effective therapies (Ostrom et al., 2017). It is necessary to find new prognostic biomarkers and therapeutic strategies. In recent years, increasing evidence suggests that RNA modifications, especially m^6^A and m^5^C, play a crucial role in tumorigenesis, and the corresponding regulatory enzymes are potential candidates of prognostic biomarkers and therapeutic targets (Wang P. et al., 2020; Lin et al., 2020). In this line, Ψ synthase genes have been found to be involved in tumor progression and prognosis (Jana et al., 2017; Elsharawy et al., 2020; Du et al., 2021). In current study, we demonstrated the value of Ψ synthase genes in prediction of the prognosis of gliomas, and constructed a novel five-gene-based prognostic model (Figures 2–4).

Since a large amount of Ψ can be detected in urine of various malignant neoplasms, the level of Ψ in urine has been proposed as a potential tumor marker (Nombela et al., 2021). The level of Ψ in urine mainly depends on the metabolism and turnover of RNA, thus dysregulation of Ψ synthases may cause Ψ to accumulate in urine (Gehrke et al., 1979). Although the relationship between elevated Ψ level and imbalance of pseudouridine synthases remains to be elucidated, performing transcriptomic analysis of Ψ synthases and evaluating Ψ levels in the blood, urine or tissue of patients in relevant clinical characteristics such as WHO grade, IDH status, may greatly aid in identifying the differences between gliomas of various clinicopathological parameters, and potentially promote the diagnosis and treatment of glioma. Notably, the DKC1 inhibitor pyrazofurin and the MEK1/2 inhibitor trametinib can synergistically suppress colorectal cancer growth, suggesting the Ψ synthases are very promising therapeutic targets for cancer (Kan et al., 2021). 5-Fluorouracil (5-FU), the most widely used fluorinated pyrimidine in cancer treatment (Gmeiner, 2020), has been shown to inhibit pseudouridine synthases. Regardless of whether the related cytotoxicity of 5-FU is due to the overall decrease of RNA pseudouridylation or the loss of specific modified RNAs, evaluating the expression model of Ψ synthases may contribute to accurate application of 5-FU in cancer treatment and improve the prognosis. For glioma, small-molecule inhibitors that can cross the blood-brain barrier and specifically target Ψ synthases urgently need to be developed.

Among all of the post-transcriptional modifications identified on RNA molecules, Ψ is the most abundant modification (Charette and Gray, 2000; Ge and Yu, 2013). Pseudouridylation in the 3′-UTRs of mRNA may serve as a key factor in stabilization of mRNA (Kierzek et al., 2014; Schwartz et al., 2014; He et al., 2021). In addition, pseudouridylation fine-tunes the effects of codon bias to affect translation fidelity and efficiency (Karijolich and Yu, 2011; Karijolich et al., 2015; He et al., 2021). PUS1, PUS7, DKC1 and TRUB1 have been reported to modify pseudouridylation in mRNA (Li et al., 2015). In current study, the prognostic model was constructed based on expression of these Ψ synthase genes and RPUSD1 (Figure 4). The Ψ synthase genes might shape a new component in the signature of transcriptomic landscapes of gliomas (Figure 6).

Intriguingly, the up-regulated genes were significantly enriched in the immune-related signaling pathways, including the regulation of immune effector process, the regulation of T cell activation, the regulation of cell adhesion, and the mononuclear cell proliferation signaling pathway (Figures 6C,D). Therefore, the relationship of Ψ modification and landscape of immune microenvironment in gliomas was subsequently analyzed based on the expression data (Figures 7, 8). Consistent with previous studies, the immune scores, matrix scores, and ESTIMATE scores of high-risk gliomas were higher (Figure 7), but the immunophenoscores were lower (Supplementary Figure S5), indicating that the immune microenvironment had undergo significant suppressive changes (Fu et al., 2020a; Fu et al., 2020b). To characterize whether the risk scores were associated with the suppressive immunophenotype, an immune signature containing 39 immunosuppressive genes were analyzed. As expected, almost all of the immunosuppressive genes were up-regulated in the high-risk group, including the checkpoint genes such as PD-1, PD-L1 and CTLA4 (Figures 7E,F). Moreover, decreased infiltration of monocytes, NK cell activation and T cell CD4^+^ naive, as well as increased infiltrating of M0 and Tregs were found in the tumor microenvironment, which may contribute to the characteristic of local immune suppression in the high-risk group (Figure 8). These findings provide a novel insight into the relationship between Ψ modification and immunosuppressive microenvironment.

An association of the inflammatory molecules, CASP4 and S100A11, with the immune cell infiltration (including M0, monocytes, NK cell activation, T cell CD4^+^ naive and Tregs) was identified in TME through WGCNA analysis. In addition, their expression levels may be regulated by the five prognostic Ψ synthase genes, especially TRUB1 (Figure 10, Supplementary Figure S8, 9). CASP4 (Caspase-4) is the key molecule in the noncanonical inflammasome pathway, which can result in inflammatory cell death (pyroptosis) via cleavage of gasdermin D in monocytes and macrophages, accompanied with release of inflammatory cytokines (Schmid-Burgk et al., 2015). S100A11 is a member of S100 protein family (S100s), which participates in a variety of physiological and pathological processes, including inflammation, cell proliferation, apoptosis and cancer development (Zheng et al., 2021). S100A11 can induce chemokine response and regulate monocyte recruitment *in vivo*, but its release depends on activation of caspase (Safronova et al., 2019). Whether CASP4 (Caspase-4) and S100A11 act synergistically in gliomas remains unclear. Tumor cells may promote the inflammatory status by releasing a wide range of cytokines (Coussens and Werb, 2002; Sharma and Kanneganti, 2021). Chronic inflammation at the local and/or systemic level contributes to tumor pathobiology, progression, metastasis and drug resistance by reprogramming tumor cells and reorganizing the tumor immune microenvironment (Faria et al., 2021). Up-regulation of CASP4 and S100A11 may contribute to the inflammatory status in the tumor immune microenvironment for the proliferation, differentiation and survival of tumor cells. However, whether Ψ synthase genes can directly regulate the expression of CASP4 and S100A11, and whether the abnormal expression of CASP4 and S100A11 will affect the abundance of immune cell infiltration still requires experimental research.

## Data Availability

The datasets presented in this study can be found in online repositories. The names of the repository/repositories and accession number(s) can be found in the article/Supplementary Material.
