# Supplementary figures and images for: Gene Expression-Based Predication of RNA Pseudouridine Modification in Tumor Microenvironment and Prognosis of Glioma Patients
Source: Front Cell Dev Biol. 2022 Jan 18;9:727595. doi: 10.3389/fcell.2021.727595 (PMC8804349; doi:10.3389/fcell.2021.727595)

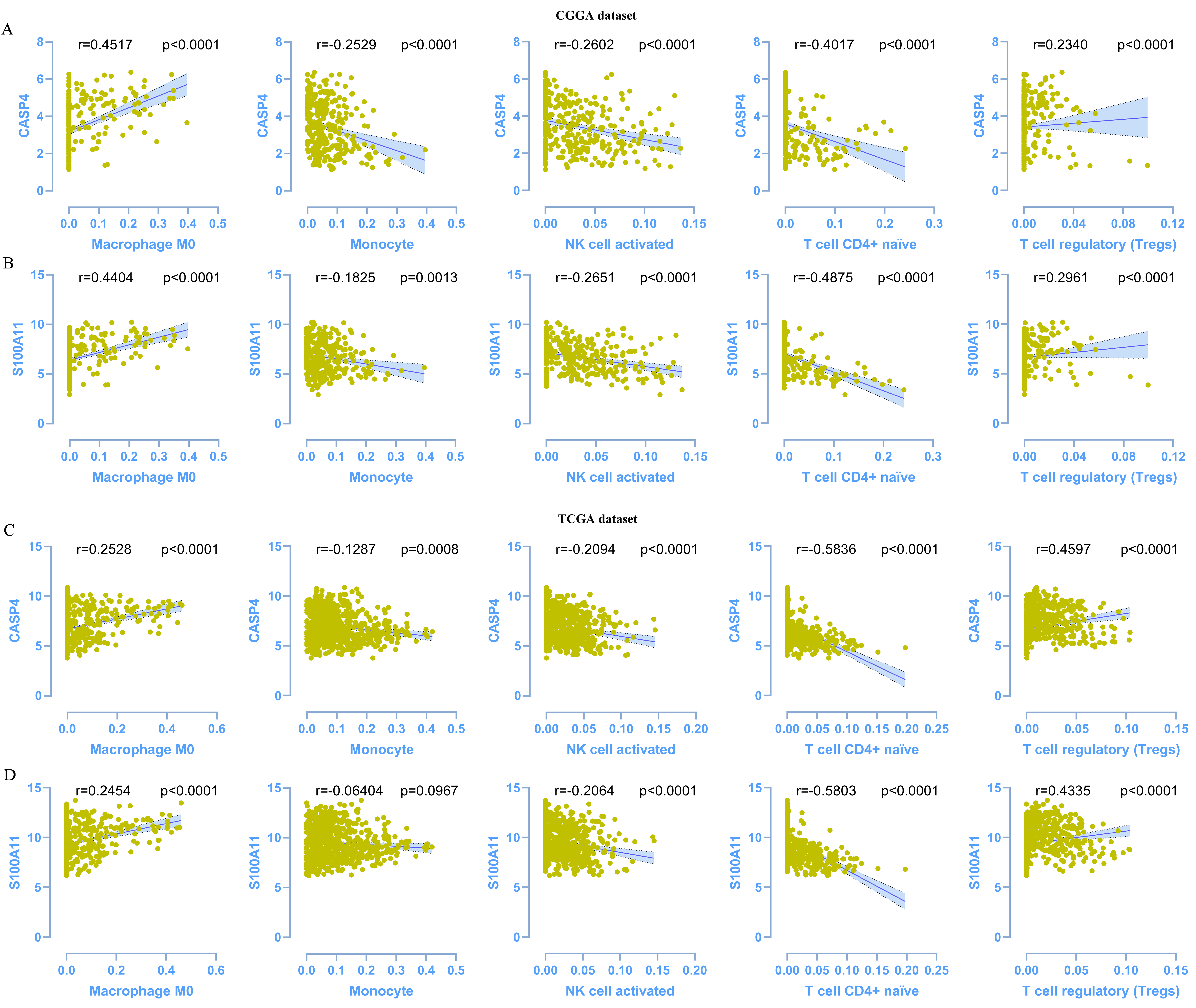

Supplement: Supplementary file 1 [file Image6.TIF]

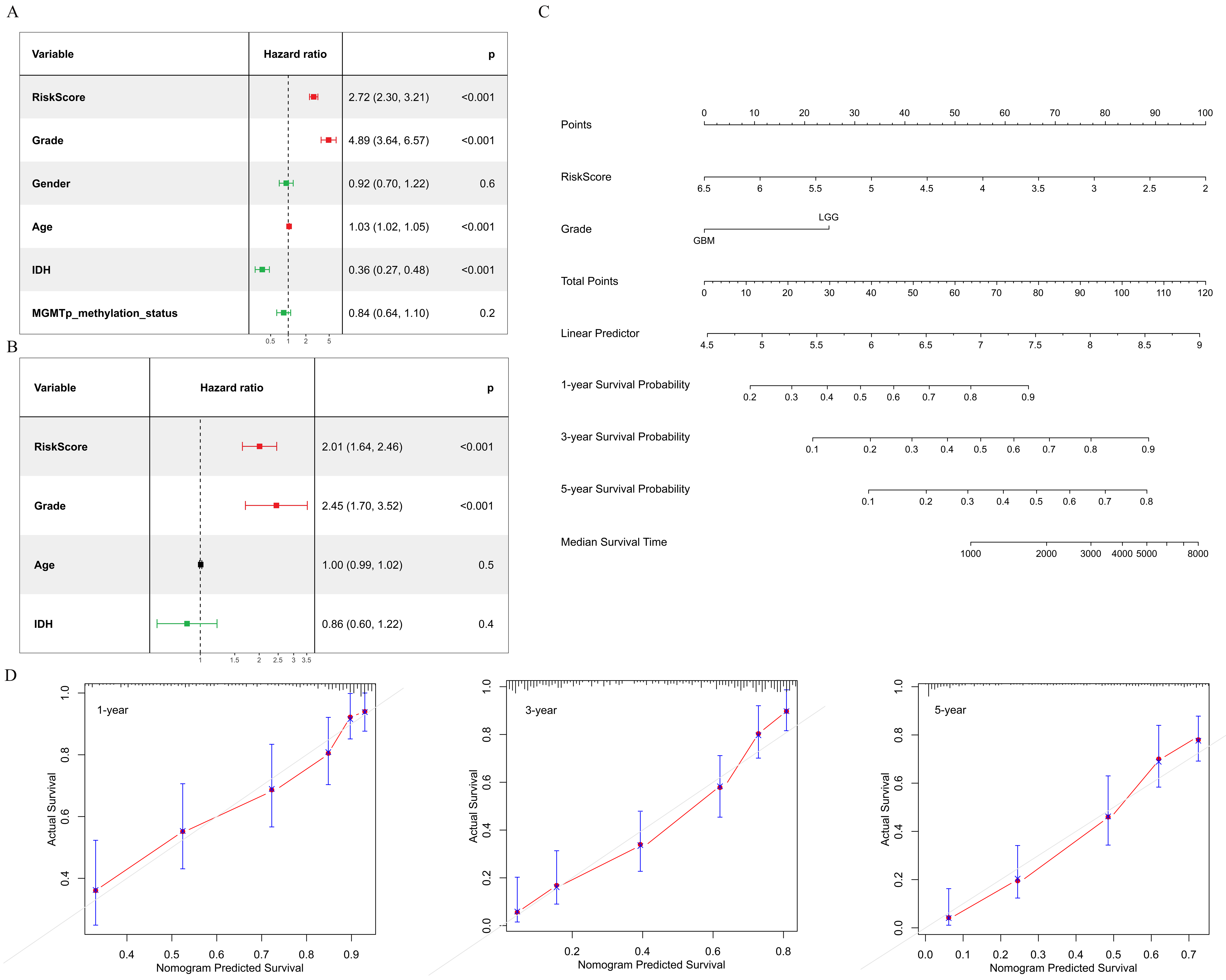

Supplement: Supplementary file 2 [file Image3.TIF]

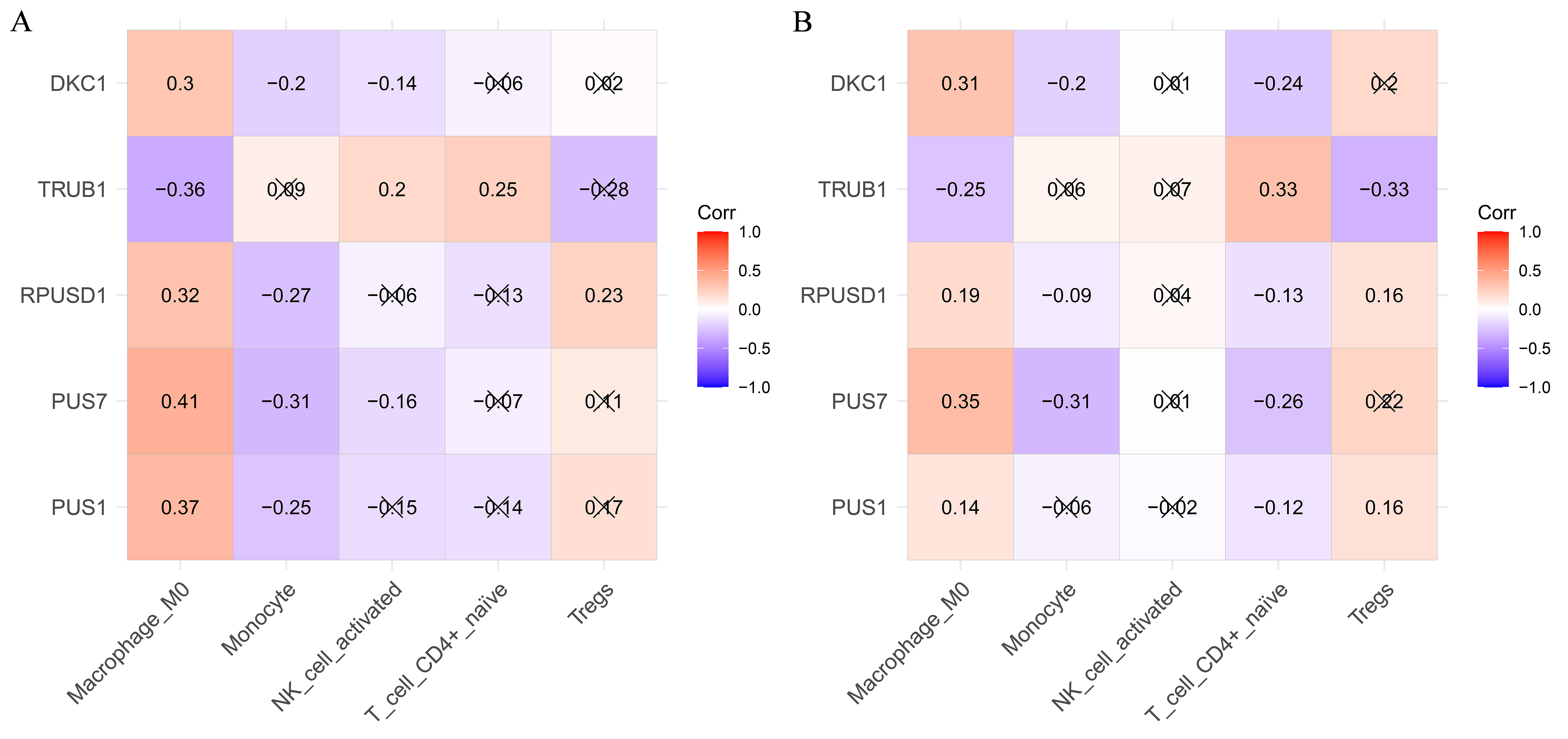

Supplement: Supplementary file 3 [file Image4.TIF]

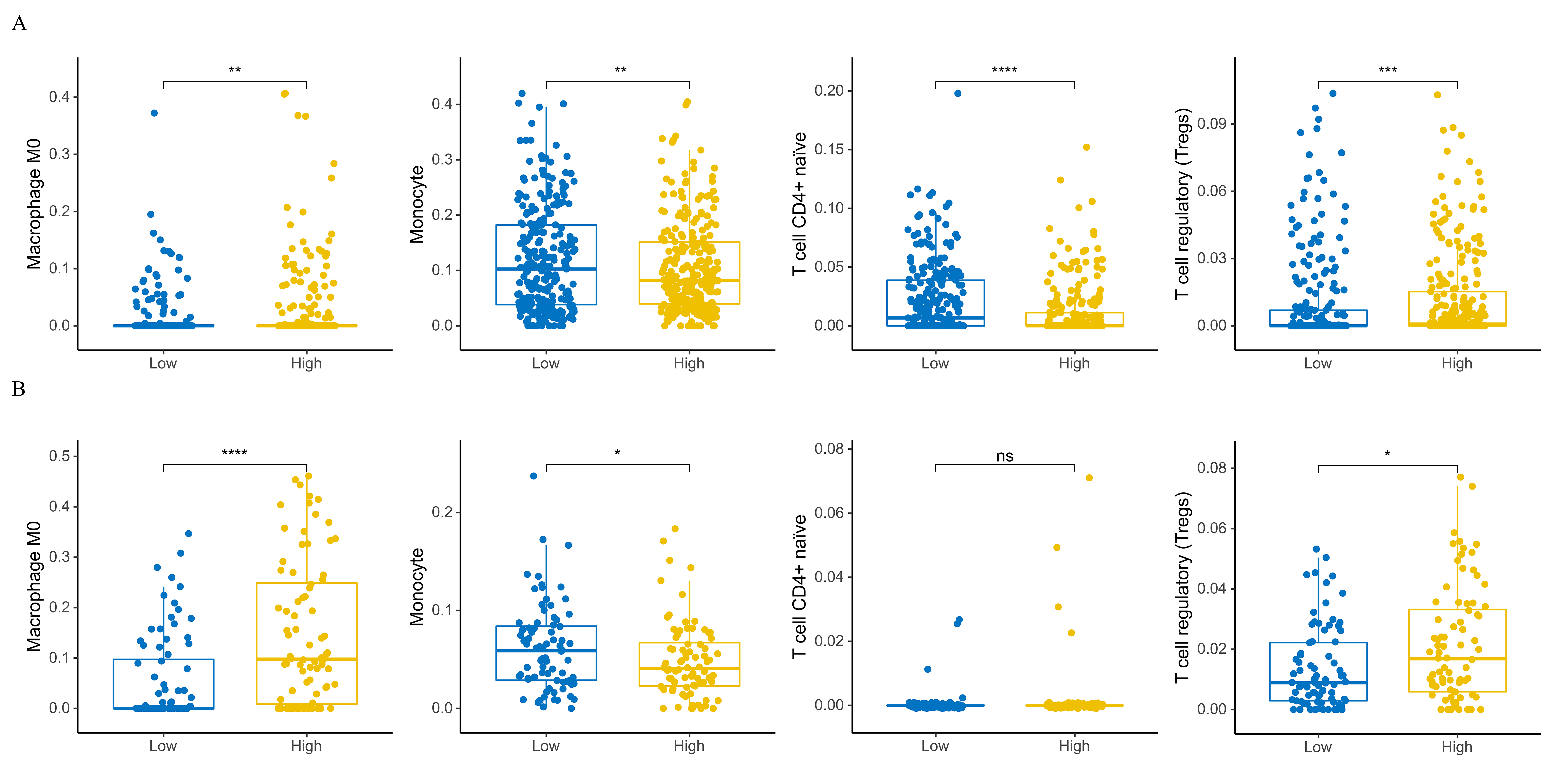

Supplement: Supplementary file 4 [file Image9.TIF]

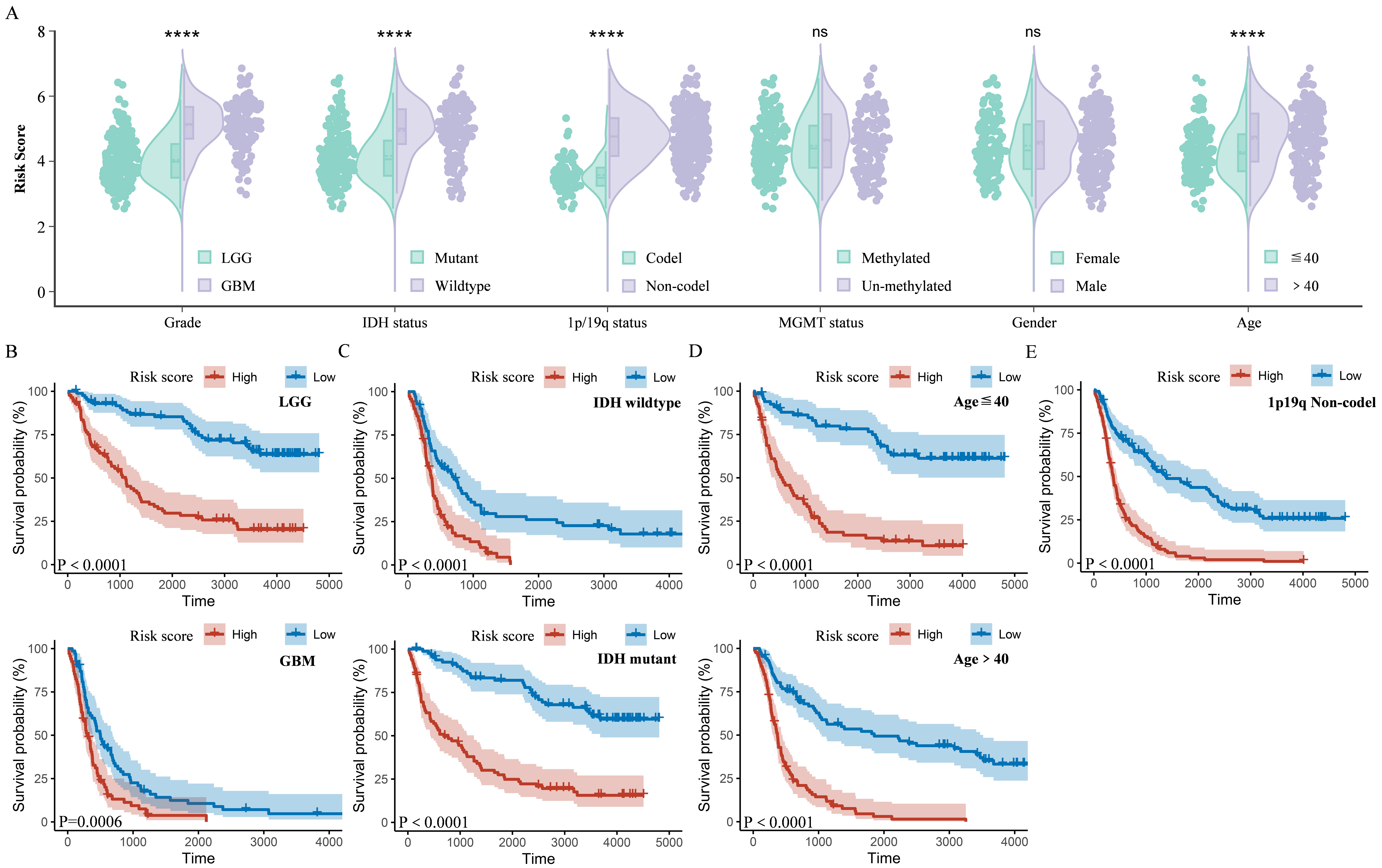

Supplement: Supplementary file 5 [file Image2.TIF]

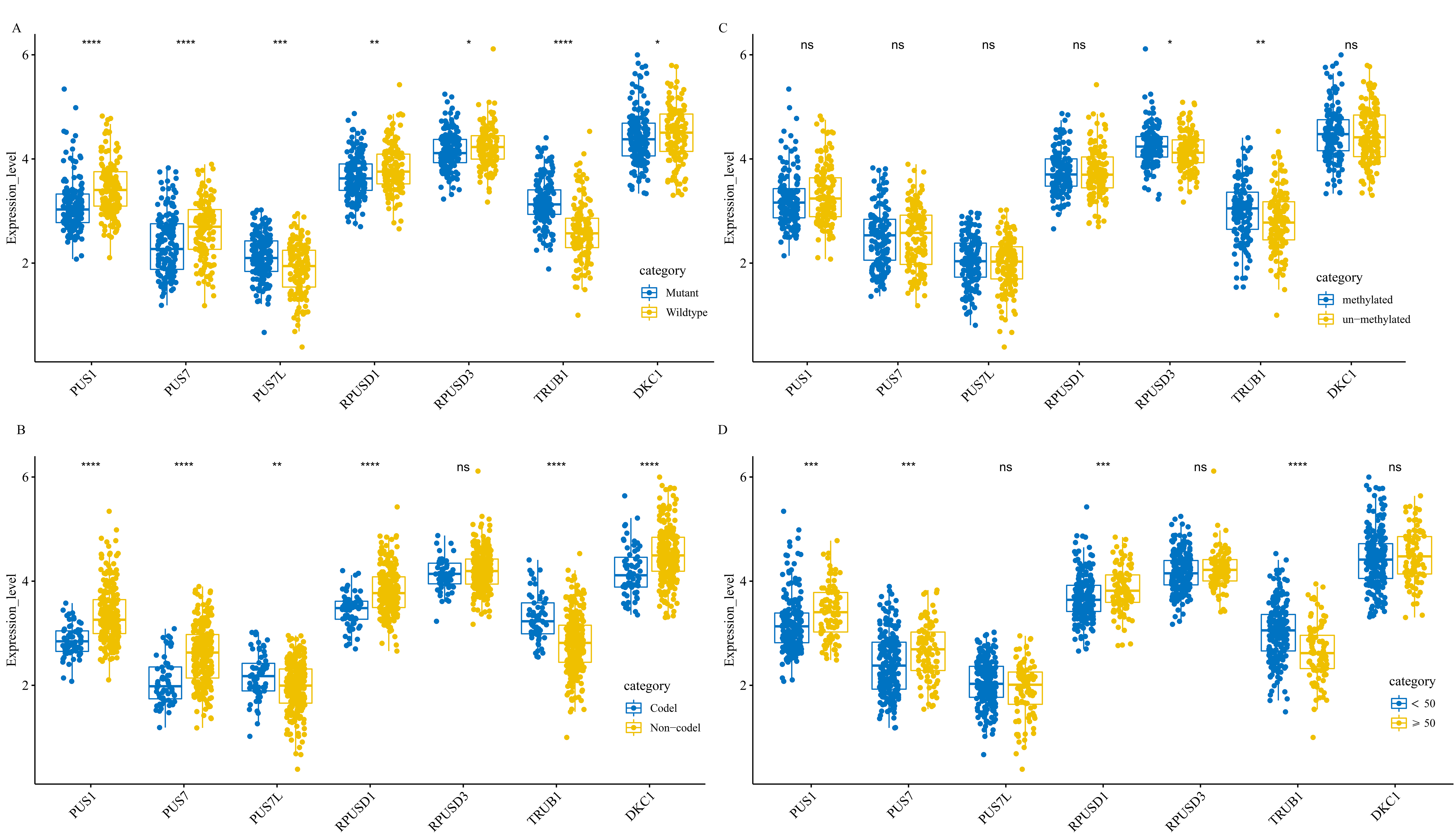

Supplement: Supplementary file 6 [file Image1.TIF]

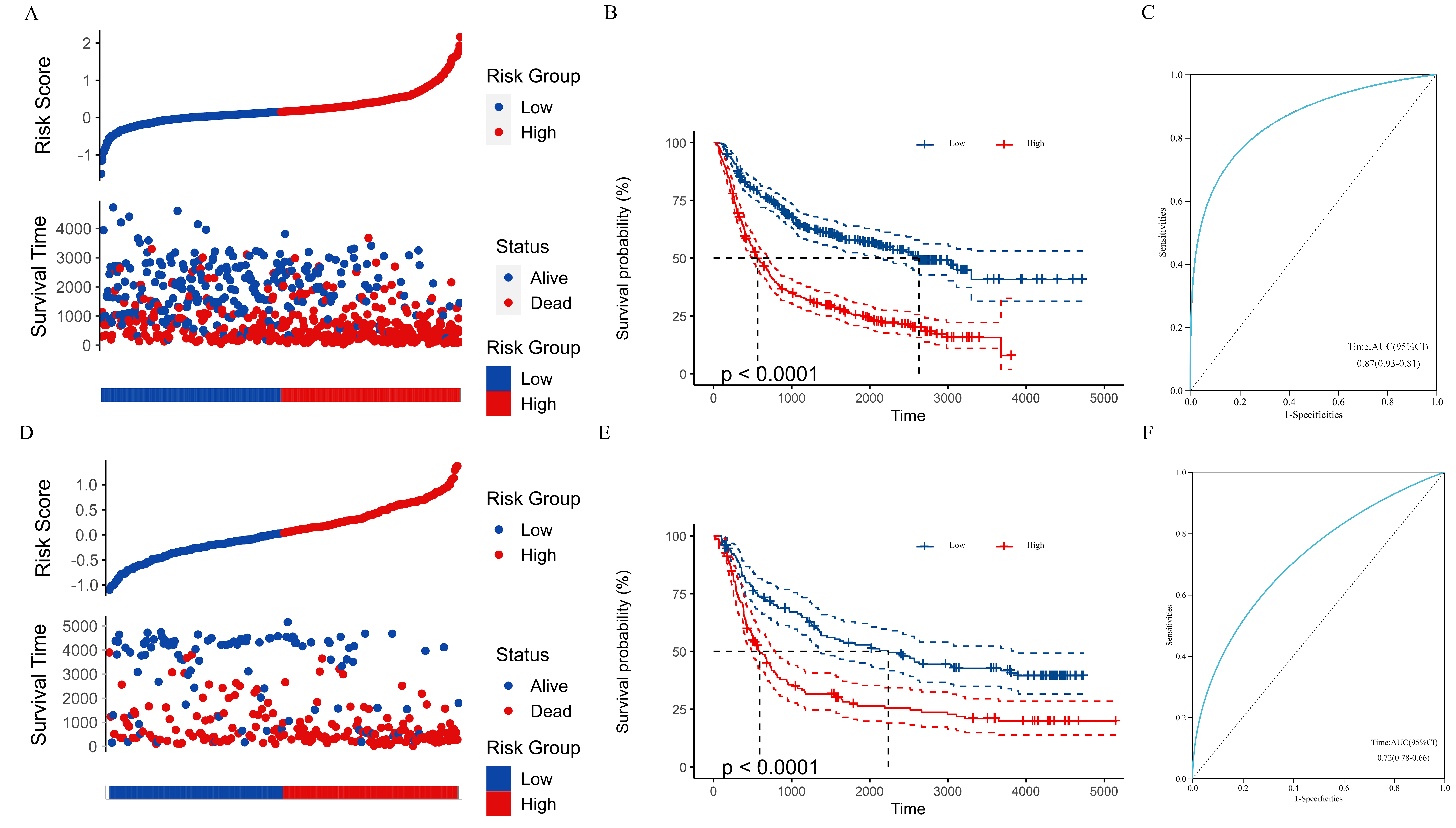

Supplement: Supplementary file 7 [file Image7.TIF]

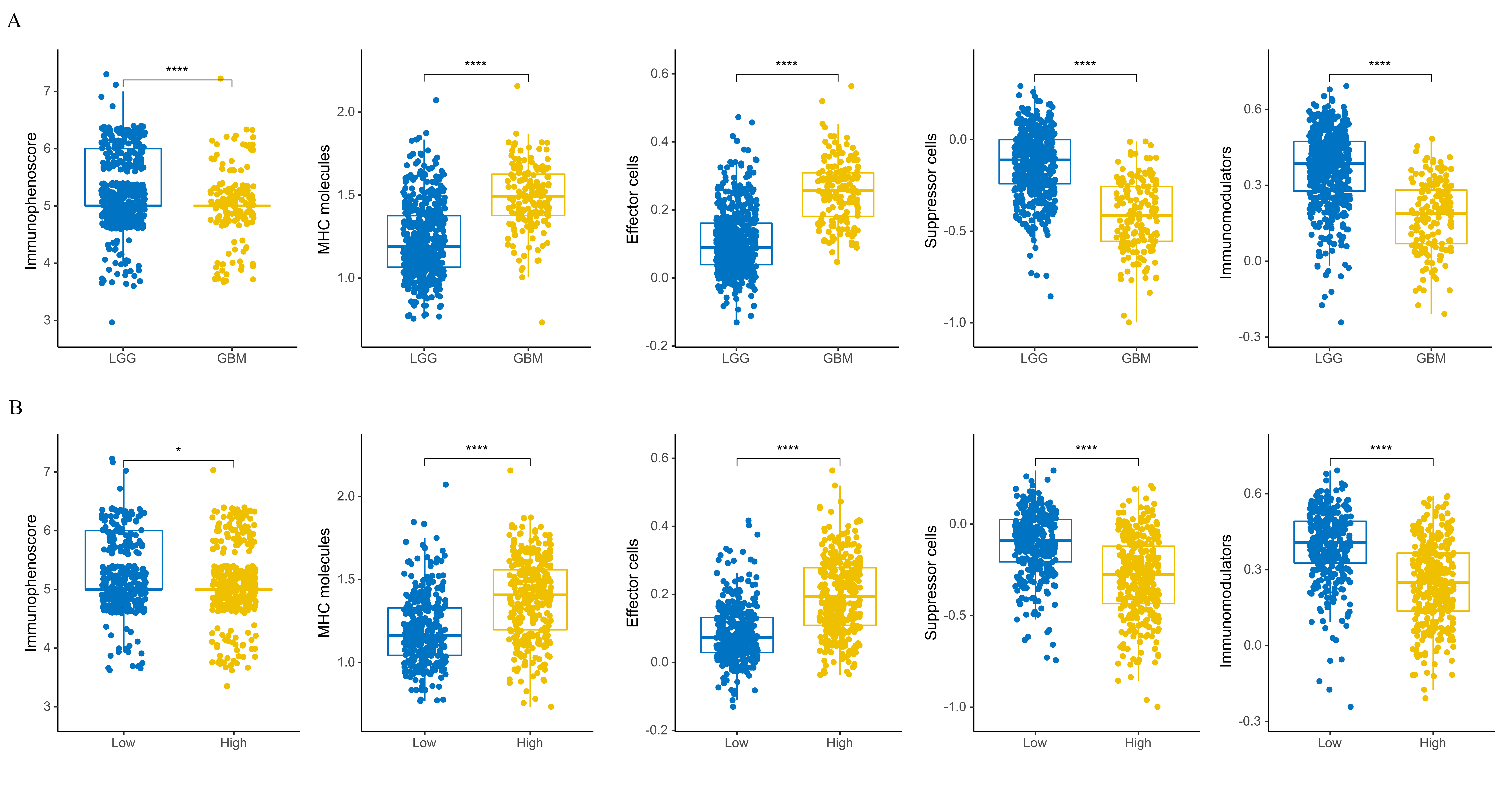

Supplement: Supplementary file 9 [file Image8.TIF]

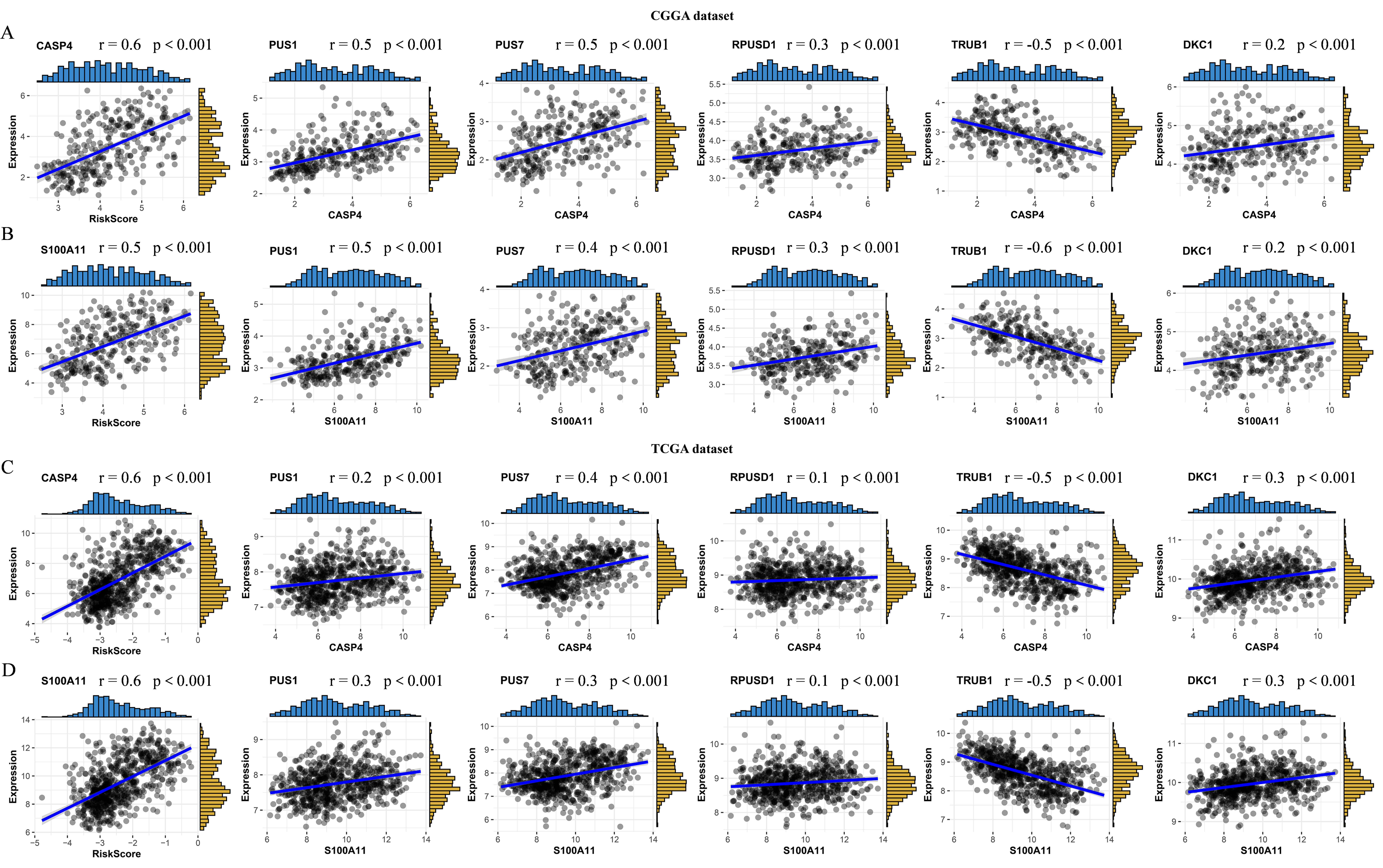

Supplement: Supplementary file 10 [file Image5.TIF]
